# Diffusion tensor imaging in Alzheimer's disease: insights into the limbic-diencephalic network and methodological considerations

**DOI:** 10.3389/fnagi.2014.00266

**Published:** 2014-10-02

**Authors:** Julio Acosta-Cabronero, Peter J. Nestor

**Affiliations:** Brain Plasticity and Neurodegeneration Group, German Center for Neurodegenerative Diseases (DZNE)Magdeburg, Germany

**Keywords:** neurodegenerative diseases, Alzheimer's disease neurobiology, axonal loss, *circuit of Papez*, long association tracts, splenium, DTI criteria, Alzheimer's disease biomarkers

## Abstract

Glucose hypometabolism and gray matter atrophy are well known consequences of Alzheimer's disease (AD). Studies using these measures have shown that the earliest clinical stages, in which memory impairment is a relatively isolated feature, are associated with degeneration in an apparently remote group of areas—mesial temporal lobe (MTL), diencephalic structures such as anterior thalamus and mammillary bodies, and posterior cingulate. These sites are thought to be strongly anatomically inter-connected via a limbic-diencephalic network. Diffusion tensor imaging or DTI—an imaging technique capable of probing white matter tissue microstructure—has recently confirmed degeneration of the white matter connections of the limbic-diencephalic network in AD by way of an unbiased analysis strategy known as tract-based spatial statistics (TBSS). The present review contextualizes the relevance of these findings, in which the fornix is likely to play a fundamental role in linking MTL and diencephalon. An interesting by-product of this work has been in showing that alterations in diffusion behavior are complex in AD—while early studies tended to focus on fractional anisotropy, recent work has highlighted that this measure is not the most sensitive to early changes. Finally, this review will discuss in detail several technical aspects of DTI both in terms of image acquisition and TBSS analysis as both of these factors have important implications to ensure reliable observations are made that inform understanding of neurodegenerative diseases.

## Introduction

Alzheimer's disease (AD) is characterized, histopathologically, by amyloid deposition and neurofibrillary tangles (composed of hyperphosphorylated tau); these features occur in somewhat topographically distinct distributions in the brain. The ultimate outcome of AD is neuronal loss though exactly how histopathological features interact with each other and how they relate, in turn, to neuronal degeneration remains rather unclear. It seems reasonable to assume, nonetheless, that neuronal—and synaptic—loss is critical to the development of cognitive impairment. To date, therapeutic attempts to slow the course of AD have targeted the histopathology. A possible alternative, or even complimentary approach, might be to understand what makes some neuronal populations in the central nervous system more vulnerable to degeneration than others with a view to finding ways to make neurons less vulnerable to pathological insult—regardless of what that pathological insult might be. Such an approach requires a precise understanding of the spread of neurodegeneration and this can only be achieved by studies in humans rather than disease models.

Historically, *in vivo* work to understand the landscape of neurodegeneration in AD has focused on atrophy detection using T_1_-weighted magnetic resonance imaging (MRI) and metabolic studies using (^18^F)-2-fluoro-deoxy-D-glucose positron emission tomography (FDG-PET)—the latter being primarily a marker for synaptic loss. Both of these modalities typically focus on changes in gray matter (GM) and have tended to show what, at first glance, appear to be different profiles of degeneration (Ishii et al., 2005). Structural MRI has been good at highlighting mesial temporal lobe (MTL) atrophy (Du et al., 2001; Jack et al., 2002, 2004), while FDG-PET has typically highlighted early changes in posterior association cortex (Minoshima et al., 1995) with the posterior cingulate region in particular appearing as the first hypometabolic region in very early symptomatic AD (Minoshima et al., 1997; Nestor et al., 2003a). This apparent discrepancy between structural MRI and FDG-PET may well, however, be technical. For instance, much of the work on MTL atrophy derives from region-of-interest (ROI) studies that did not examine for atrophy elsewhere. Where whole brain analyses were conducted, most used the voxel-based morphometry (VBM) technique (Ashburner and Friston, 2000) though recent evidence highlights that although this method is good at identifying MTL atrophy, it is relatively insensitive to atrophy in the isocortical ribbon (Diaz-De-Grenu et al., 2014); in contrast, using the Freesurfer's cortical thickness method (Dale et al., 1999; Fischl et al., 1999), atrophy of the cortical ribbon in posterior association cortex emerges in a pattern highly reminiscent of that seen with FDG-PET (Diaz-De-Grenu et al., 2014). Similarly, although ROI studies in the cortical ribbon are rare, manual volumetry of the posterior cingulate region in MCI-stage AD has confirmed comparable degrees of atrophy to that seen in the hippocampus (Choo et al., 2010; Pengas et al., 2010). Looking at the apparent discrepancy in these imaging modalities from the FDG-PET side, voxel-based analysis (VBA) appears relatively insensitive to MTL changes in early AD; however, using MRI-derived ROIs to calculate cerebral metabolic rates (i.e., quantified imaging), FDG-PET identified significant hypometabolism not only in the posterior cingulate but also the MTL, as well as anterior thalamus and mammillary bodies (Nestor et al., 2003b). This “limbic-diencephalic” network, therefore, appears to be the correlate of very early symptomatic AD when memory impairment is a relatively isolated feature. It is interesting, therefore, that these network structures—MTL, posterior cingulate, anterior thalamus and mammillary bodies—which are connected through the *circuit of Papez* (Papez, 1937), have all been individually implicated from focal lesions in human amnesia.

The hypothesis that these structures are degenerating in concert predicts that white matter (WM) projections between these areas—such as, for instance the fornix, which links the MTL with the diencephalon—should also show signs of degeneration. To test such hypotheses as well as to understand how degeneration in AD may impact on areas remote from GM degeneration more generally, the relatively more recent technique of diffusion tensor MRI offers considerable promise. Diffusion MRI enables mapping of WM microstructure alterations in development, aging and neurological disorders, and has therefore become an important tool in the study neurodegeneration. Parenchymal WM is composed of bundles of axons (or fiber tracts) that interconnect GM areas. The diameter of neuronal fibers is well below MRI resolution but the technique can be sensitized to measure the displacement of water molecules as a surrogate marker of tract integrity. Axonal membranes, myelin sheaths and cytoskeletal constituents such as microtubules and neurofilaments are long structures that may hinder water diffusion preferentially perpendicular to their length; this phenomenon enables MRI to detect abnormalities caused by neurodegenerative diseases such as loss of fibers, demyelination, damage within fibers or to support tissue around them (Beaulieu, 2002). The technique remains fairly new and there has been a relatively steep learning curve in terms of understanding and interpreting diffusion tensor imaging (DTI) (Alger, 2012; Winston, 2012); this learning process is far from over. What is clear at this time is that technical factors—both in terms of acquisition parameters and analysis methods—play a more important role in terms of generating spurious findings compared to older modalities such as structural T_1_-weighted imaging and FDG-PET. The outcome being that the DTI literature in AD can come across as a confusing jumble of inconsistent abnormalities—an unsurprising observation considering DTI's vulnerability to spurious results when suboptimal experimental designs are employed. The prescription of DTI acquisitions with fewer-than-recommended diffusion-encoding directions, low *b*-values or thick slices; the study of mild cognitive impairment (MCI) cohorts without clinical outcome and the detrimental effect of Gaussian smoothing in post-processing pipelines are the most likely contributors to such inconsistencies.

It is crucial therefore that clinicians and researchers have some understanding of the pitfalls that can arise from inadequate methods in order to interpret what have often been rather contradictory results in DTI studies. This review will focus on what we have learned from DTI in AD to date, but also will go into some detail in explaining the methodological issues that can cause problems for DTI studies.

## The physical basis of DTI

The present section is not intended as a comprehensive description of DTI theory—for that, extensive reviews can be found elsewhere (Kingsley, 2006a,b,c). It aims, however, to provide an intuitive understanding of the method and an appreciation of the possible tensor dynamics that can arise with neurodegeneration; these are important theoretical considerations because diffusion, as measured with the “single tensor” model (Basser et al., 1994b), is based on a number of assumptions that lead to certain limitations; limitations that have been extensively discussed in the diffusion MRI literature (Jones and Cercignani, 2010; Jones et al., 2013b), but that are often overlooked in clinical studies. Nevertheless, DTI is a powerful technique to study neurodegenerative diseases; thus, an overview of its theoretical foundations will also enable a more clear understanding of the discoveries that have been made to date in AD. The theoretical underpinning of DTI can, however, seem impenetrable for non-specialists even when most of the background mathematics is omitted, as is the case in this section. For readers, therefore, who might find the physics described in this section too daunting, we suggest simply focusing on the Figures and their captions; and with a brief understanding of what a diffusion tensor means, one can then skip on to the following section “Technical considerations for clinical DTI studies” for a discussion of the factors that influence the suitability of DTI scans for clinical studies.

**Figure 1:**
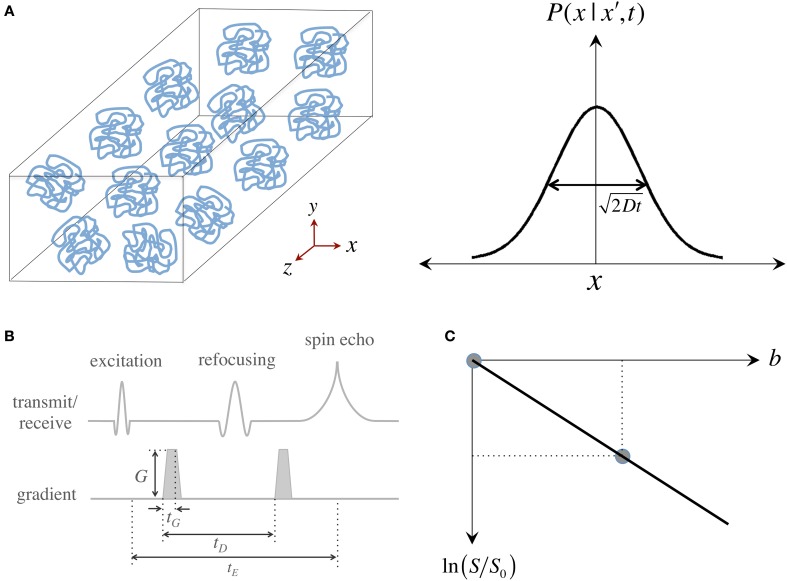
**Measurement of the “self-diffusion” coefficient with magnetic resonance**. **(A)** Self-diffusion is a special case of diffusion that describes the relentless motion of microscopic particles in a substance. Such motion is random—it can be described by a “Gaussian” distribution whose width determines how far particles travel on average after some time; the important thing to remember is that only time, the “diffusion time,” and the “self-diffusion coefficient,” *D*, modulate this width. **(B)** Illustration of a magnetic resonance pulse sequence that is sensitive to self-diffusion: (i) an “excitation pulse” gives some energy to water protons; (ii) then the first “gradient” *encodes* where they are; (iii) and some (diffusion) time later, a second gradient *decodes* their position by giving them a “signal penalty” according to how far they have moved away from their original position; (iv) as a result of this penalty, protons that have moved furthest, return the least signal. The overall signal attenuation is a function of *D* and the gradient characteristics—i.e., intensity (*G*), duration (*t_G_*) and diffusion time (*t_D_*)—usually conglomerated into a single term known as the “*b*-value.” **(C)** Visual representation of the linear fit required to quantify *D* using two signals: a diffusion weighted measurement (i.e., with a non-zero *b*-value), *S*, that must be normalized by a measurement without diffusion weighting, *S*_0_ (also often called b_0_); *D* can be inferred as the “negative” of the slope.

Brownian motion, also known as “self-diffusion,” is a physical phenomenon arising from matter's intrinsic thermal energy that leads to pseudo-random kinetic fluctuations. Such molecular thermal motion is named after Robert Brown—a Scottish botanist—who first observed such behavior in grains of pollen suspended in water (Brown, 1828). A full mathematical description emerged a few decades later when Adolf Fick—a physiologist studying mass transport in saline solutions—proposed that microscopic motion could be seen as a probability density function (*pdf*) of a particle's location in space and time, i.e., as the likelihood of finding a particle in a certain place at a certain time, and then went on to predict that self-diffusion must be governed by such time-dependent probabilistic behavior (Fick, 1855). Soon after the turn of the 20th century, Albert Einstein solved the general case of Fickian diffusion to provide an analytical answer to the practical question: *how far do “free particles” travel “in average” during a time interval?* Einstein proposed a simple “random walk” model comprising a series of discrete, unrestricted, independent and uncorrelated steps, which led him to discover that for such boundary conditions, Fick's *pdf* is a Gaussian distribution whose width—a measure of average molecular displacement—is modulated by two factors: time and a parameter dependent on fluid viscosity and temperature; he figured the latter must be a self-diffusion coefficient, *D* (Einstein, 1905) (see Figure 1A).

Since the inception of “spin echoes” in 1950, the MR signal has been known to be sensitive to molecular thermal motion (Hahn, 1950; Carr and Purcell, 1954; Torrey, 1956), which hereafter will be assumed to be that of hydrogen protons in water molecules within human brain tissue. Applying Einstein's relationship, it was determined that “free diffusion” under the effect of a steady magnetic field “gradient” attenuates the MR signal. This was first observed because field gradients (i.e., gradual increments or decrements in magnetic field strength) occur naturally when an experimental sample is introduced into the uniform magnetic field of a magnet and disturbs it. Magnetic field gradients, however, can be applied artificially to vary the main field gradually along one direction; such gradual field change, in essence, labels protons according to their spatial position. Stronger gradients, therefore, have the capability of giving a wider range of spatial signatures that result in higher image resolutions (if we refer to imaging gradients) or finer sensitivity to motion (if we refer to diffusion MRI). It was soon realized, however, that measuring self-diffusion through the application of sustained gradients was inconvenient and largely ineffective. Then in 1965, Stejskal and Tanner proposed that the amount of diffusion weighting in the MR signal could be finely controlled with a pair of identical, short-lived, magnetic field gradients (Stejskal and Tanner, 1965) (see Figure 1B). Pulsed gradients sequentially label, and then unlabel, protons according to their position; with the result that if a proton moves to a different location after a “diffusion time”—i.e., when the second gradient is applied—, the proton gets assigned a wrong label and returns less signal according to how far it has moved. This principle—the basis for most diffusion MRI acquisitions today—results in an overall signal intensity attenuation due to free diffusion that depends on the self-diffusion coefficient, *D*, and the gradient characteristics. For simplicity, and because in MRI there is a complex interaction between diffusion and imaging gradients, the overall effect of motion-sensitizing gradients is often synthesized into a so-called “*b*-value.” This is convenient because the signal attenuation due to diffusion can be expressed simply by an exponential function of *D* and *b*, i.e., *S* ∝ exp (−*bD*). As in any spin echo experiment, however, the signal also decays due to transverse relaxation (T_2_) effects; thus, for the signal to depend only on the effects of water mobility, it must be normalized by a reference measurement without diffusion weighting, *S*_0_, also known in DTI jargon as a *“*b_0_ scan.” Two measurements, therefore, are sufficient to infer a diffusion coefficient as: *D* = −ln (*S/S*_0_)/*b*; or in visual representation terms, that *D* is the negative of the slope connecting the two data points in Figure 1C.

The information that a Stejskal-Tanner experiment provides, however, is limited because the positions of resonant protons can only be encoded along the direction of the applied field gradient; thus, *D* only reflects the effect of self-diffusion along that specific spatial dimension. This is enough to characterize an isotropic medium, e.g., if measuring water's self-diffusion in a bucket, because diffusion measured in a given dimension will be same as that of every other dimension, but to probe a complex system with multiple, rotationally variant diffusion behaviors such as biological tissue one needs, in theory, an infinite number of observations with sensitizing gradients along an infinite number of diffusion orientations.

In an attempt to capture such directional dependency while keeping experimental requirements feasible, Basser et al. proposed the generalization of Einstein's equation by extending the Gaussian *pdf* idea to a second order, symmetric, definite-positive covariance matrix, **D**, and this is the diffusion tensor (Basser et al., 1994a):

(1) $$D=\left(\begin{array}{ccc} D_{xx} & D_{xy} & D_{xz}\\ D_{yx} & D_{yy} & D_{yz}\\ D_{zx} & D_{zy} & D_{zz} \end{array}\right)$$

**Figure 2:**
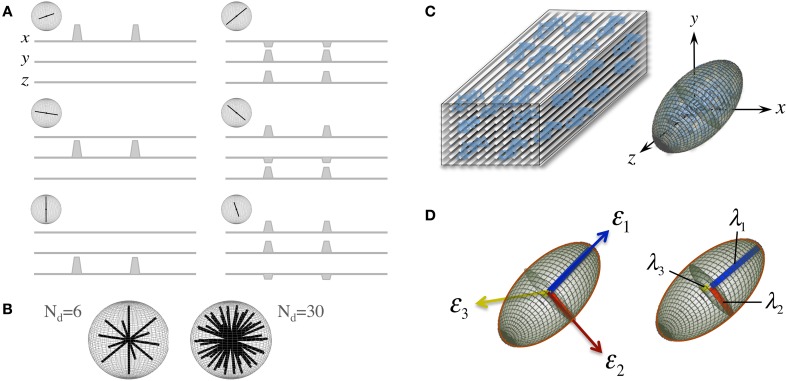
**The “diffusion tensor.” (A)** Six combinations of gradients applied with different intensities along three orthogonal orientations (*x*. *y*, and *z*), and a measurement without diffusion weighting, are the minimum requirements to probe diffusion in a 3D space; each non-collinear combination is called a “diffusion direction.” **(B)** A larger number of diffusion directions, N_d_, can estimate the directional dependence of microstructural restrictions to water diffusion with greater precision. **(C)** Basser et al. (1994b) proposed the formulation of a diffusion tensor (represented by an ellipsoid) to capture diffusion behaviors in 3D with a finite N_d_. **(D)** The tensor can be rotated to match the principal direction of diffusion, i.e., in white matter, the first eigenvector (ε_1_) matches the predominant fiber tract orientation; thus, the largest eigenvalue (λ_1_, known as “axial diffusivity”) quantifies water mobility along this orientation. Analogously, ε_2_ and ε_3_ define the plane perpendicular to the axonal arrangement so that λ_2_ and λ_3_ can be averaged as a “radial diffusivity” (RD). In addition, all three eigenvalues can be averaged to estimate the “mean square displacement” of water molecules—that is, the “mean diffusivity” (MD).

**D** is a reciprocal matrix with *D_xy_* = *D_yx_, etc*—i.e., it only has six “independent” scalar elements: *D_xx_, D_xy_, D_xz_, D_yy_, D_yz_*, and *D_zz_*; thus, in order to reconstruct a diffusion tensor, the minimum requirements are one *S*_0_ (*b* = 0 s/mm^2^ or b_0_) measurement and six diffusion measurements applying gradients along six non-collinear orientations (see Figure 2A)—i.e., N_d_, the number of diffusion gradient directions, must be at least 6, though N_d_ can be larger with obvious benefits (see Figure 2B). The single tensor model proposed by Basser et al. requires that all diffusion directions (b-vectors), *b*-values and *b*_0_ information (which can also be provided through multiple scans to improve stability) must be stored as a **B**-matrix, leading to a linear system of equations that can be solved for the six independent terms of **D** and for *S*_0_ efficiently across an entire imaging volume.

Another convenient feature of the diffusion tensor is that it can be expressed in its quadratic form as an ellipsoidal surface that characterizes the water displacement probability at a given diffusion time, i.e., an ellipsoid that visualizes the 3D character of water mobility; though in the present form, i.e., that in Equation (1), **D** is still rotationally variant because it depends on the principal diffusion orientation relative to the *x*. *y*, and *z* axes of the scanner or to whatever coordinate system the b-vectors were in (see Figure 2C). **D**, however, is positive and symmetric regardless of such orientation; thus, using linear algebra, it can be easily made diagonal for a specific orientation—i.e., it can be decomposed into a set of orthonormal eigenvectors and related eigenvalues (see Figure 2D):

(2) $$D=\left(\begin{array}{c} \varepsilon_{1}\\ \varepsilon_{2}\\ \varepsilon_{3} \end{array}\right)\left(\begin{array}{ccc} \lambda_{1} & 0 & 0\\ 0 & \lambda_{2} & 0\\ 0 & 0 & \lambda_{3} \end{array}\right)\left(\begin{array}{ccc} \varepsilon_{1} & \varepsilon_{2} & \varepsilon_{3} \end{array}\right).$$

In essence, the diagonalization step in Equation (2) rotates the principal axes of **D** to match the principal directions of diffusivity, leading, as a result, to a diffusion tensor that is rotationally invariant for each imaging “voxel.”

**Figure 3:**
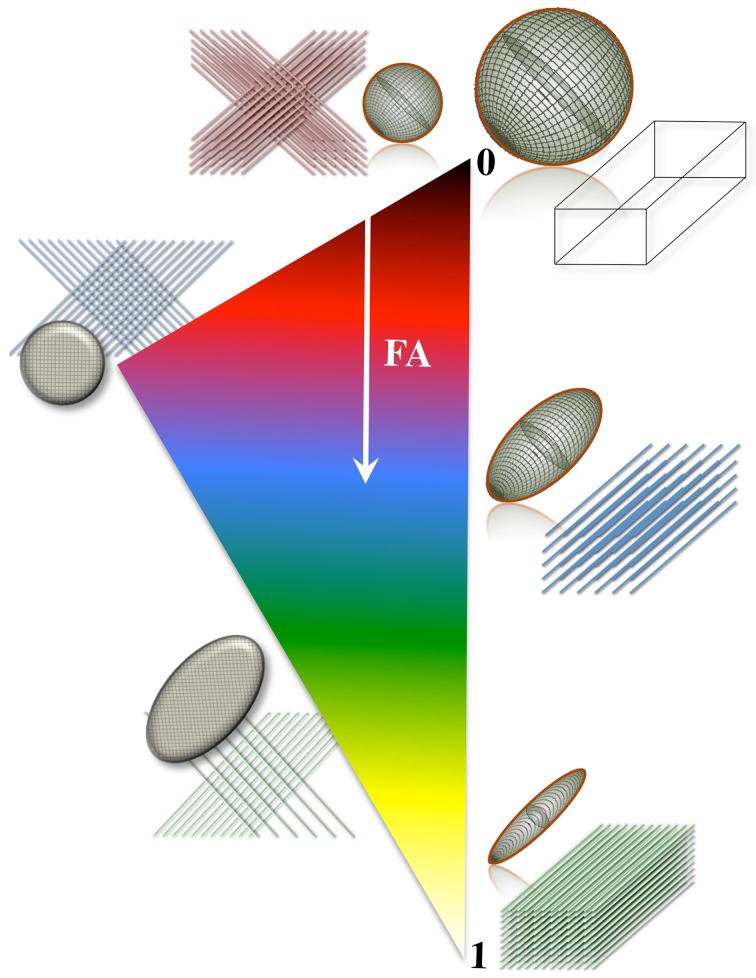
**Differential tensor behaviors in white matter**. Cell membranes are thought to be the main restricting boundary to water mobility in white matter. As such, the ratio of axial to radial diffusivities, commonly described by a metric known as “fractional anisotropy” (FA), can reflect the coherence (sometimes thus the integrity) of packed axons in white matter. FA approaches one in well-organized tracts, where the diffusion ellipsoid is elongated along the principal tract orientation; and tends to zero, in less coherent environments—i.e., in heterogeneous areas of many crossing fibers, and in gray matter or cerebrospinal fluid—, where the ellipsoid resembles a sphere. In the full complexity of white matter microstructure, however, the ellipsoid can take a wider range of geometries from that of a rugby ball to that of surfboard or a dish—with the degree of sphericity and planarity dictated primarily—but not only—by axon packing density, degree of myelination and/or the geometrical arrangement of crossing, kissing and/or splaying fiber populations.

A number of metrics can be derived from the diffusion tensor; for example, the 3D “mean square displacement” of water molecules during a diffusion time can be derived by averaging the three tensor eigenvalues to form a DTI metric known as “mean diffusivity”: MD = (λ_1_ + λ_2_ + λ_3_)/3. In addition, the rotational invariance of the diffusion tensor has clear advantages because water molecules can be generally assumed to “diffuse” due to their thermal energy more readily along the length of a uniaxial environment than perpendicular to it. It is, thus, commonly assumed when referring to, e.g., parenchymal WM, that the eigenvector, ε_1_, associated with the largest eigenvalue, λ_1_—known as “axial diffusivity”—maps the principal orientation of a fiber tract. Similarly, diffusivities along ε_2_ and ε_3_—typically averaged to form “radial diffusivity,” i.e., RD = (λ_2_ + λ_3_)/2—reflects the diffusion behavior transverse to an axonal path. Therefore, the inter-relationship between eigenvalues—i.e., between axial and radial diffusivities—contains relevant information about the geometry of the restricting microstructure. For example, in a scenario where axons are tightly packed together such as, e.g., the mid-sagittal corpus callosum, water molecules are more restricted perpendicular to the axons, hence λ_1_ >> λ_2_ ≥ λ_3_, or λ_1_ >> RD—i.e., it can be represented as a cigar-shaped diffusion ellipsoid with the long axis parallel to the axons (see Figure 3). In other regions, however, where, e.g., two tightly packed WM bundles cross, water molecules in the extra-cellular space might travel more freely across a plane, λ_1_ ≥ λ_2_ >> λ_3_, hence λ_1_ > RD—i.e., the ellipsoid adopts a planar geometry; whereas in WM areas where multiple fiber bundles cross, or in GM or cerebrospinal fluid (CSF), where water diffusion does not exhibit a preferential orientation because obstacles are randomly distributed in space or because there are no restrictions at all, the ellipsoid is largely “isotropic,” i.e., λ_1_ ≈ λ_2_ ≈ λ_3_, thus λ_1_ ≈ RD. The eccentricity of the diffusion ellipsoid, therefore, is an important property that can provide useful information about the biological tissue under investigation. This property is typically measured using the “second moment” of the diffusion tensor, because of its robust noise properties, by a metric known as fractional anisotropy (FA): $\text{FA}=\sqrt{3/2}\sqrt{\left[{\left(\lambda_{1}-\text{MD}\right)}^{2}+{\left(\lambda_{2}-\text{MD}\right)}^{2}+{\left(\lambda_{3}-\text{MD}\right)}^{2}\right]/\left(\lambda_{1}{}^{2}+\lambda_{2}{}^{2}+\lambda_{3}{}^{2}\right)}$ (Basser and Pierpaoli, 1996). FA can be seen as the relative ratio between axial and radial diffusivities, where its boundary situations are: (i) *FA* = 0 for a perfectly “ergodic” or “isotropic” behavior, i.e., when λ_1_ = RD, and (ii) *FA* = 1 for 1D molecular displacements in a 3D space, i.e., when RD is infinitesimally small relative to λ_1_. The latter behavior is the extreme case for “anisotropic” diffusion. In graphical terms (Figure 3), isotropic diffusion (*FA* = 0) is a perfect sphere because it means water can travel equally in all directions, whereas if water mobility is restricted disproportionally along one or two dimensions in space, FA moves away from zero.

**Figure 4:**
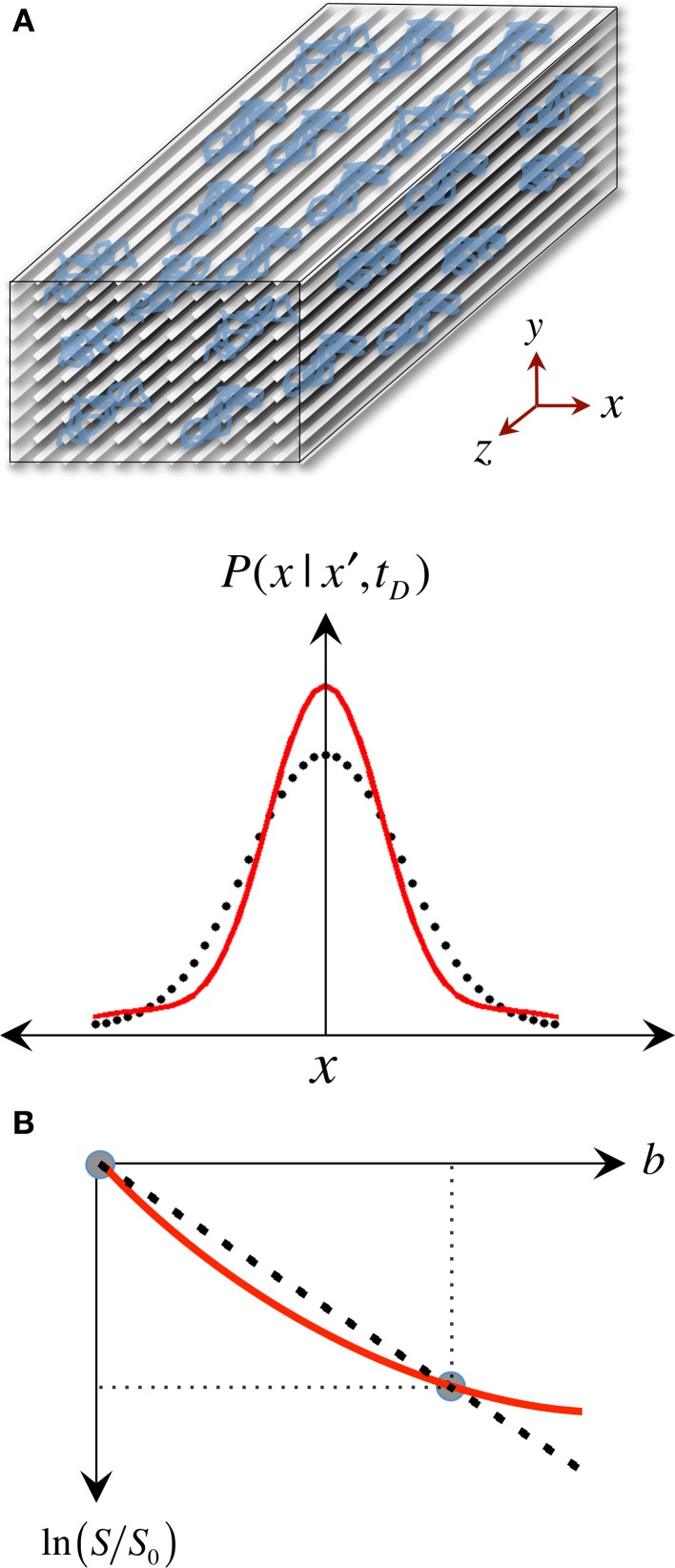
**The approximate nature of the “single tensor” model**. **(A)** “Restricted” diffusion processes such as those in white matter result in displacement probabilities that are no longer accurately described by a Gaussian “bell.” **(B)** Therefore, the assumption that the signal attenuation must have a linear relationship in a logarithmic scale with the *b*-value is no longer valid, resulting in tensor diffusivities that are “apparent” (i.e., model approximations) rather than “true” measures of restricted self-diffusion.

The pseudo-random (Gaussian) nature of unrestricted diffusional motion is, in turn, the theoretical basis supporting the single tensor model in DTI (Basser et al., 1994b); this highlights, however, one of DTI's major limitations—it is based on an idealization. As already discussed, in WM, cytoarchitectural barriers hinder and physically restrict water mobility (see Figure 4A); such nuisances to self-diffusion, therefore, impose a different set of boundary conditions to Fick's equation, making “walks” no longer random. Instead, they become correlated and dependent on the geometry of the restricting boundaries; thus leading to *pdf* departures (narrowing) from the ideal Gaussian behavior (see Figure 4A). In such scenarios, the diffusion tensor proposed by Basser et al. does not contain enough terms to account for the higher order effects in non-Gaussian molecular displacement distributions; thus, they lead to the violation of DTI's basic assumption that the signal decay due to diffusion follows a single exponential behavior. Consequently, the single tensor model must be regarded as an approximation (Figure 4B); and the three tensor-eigenvalues—if they describe restricted water motion—are not true self-diffusion coefficients, but “apparent” measures of diffusion or “diffusivities.”

**Figure 5:**
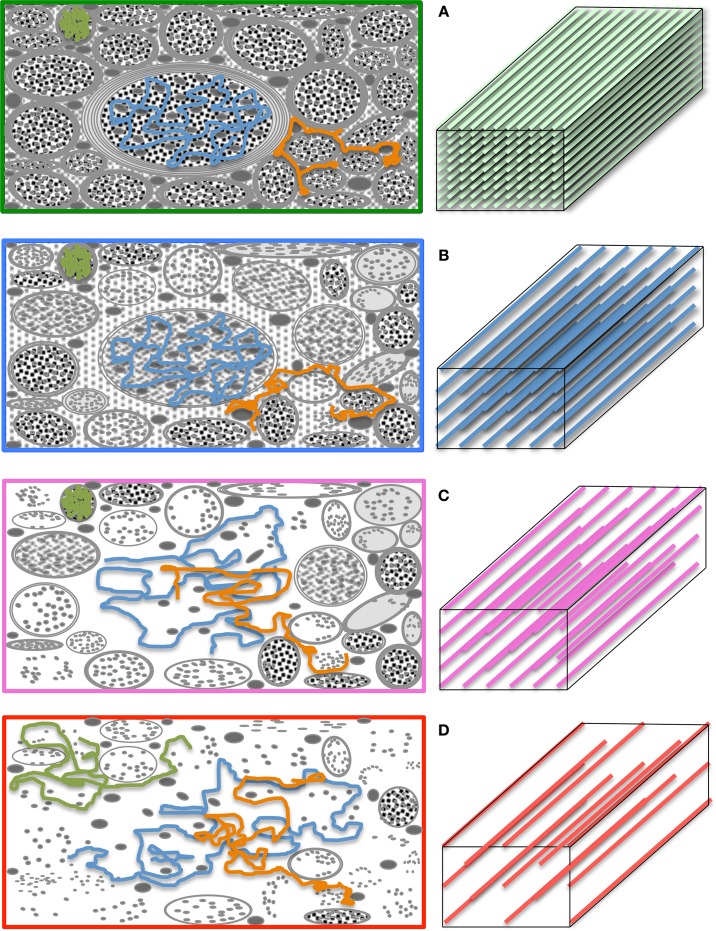
**The “diffusion regimes.” (A)** White matter is a complex—but relatively ordered— microstructure primarily composed of nerve fibers (axons) and glial cells. Axons are bundled together; their main role is to transport substances intra-cellularly through microtubules and conduct electricity to enable inter-communication between cells. The myelin sheath—a “fatty” insulating layer around the axons—facilitates such conduction. In the healthy brain, such microstructure exerts restrictive boundary conditions to water diffusion. **(B)** The exact mechanism by which microstructural damage occurs in neurodegenerative diseases is unknown but it is conceivable that, after a period of instability, demyelinative and other axon degeneration processes will lead to longer “diffusion paths.” If therefore, the diffusion time is sufficiently long and the gradients sufficiently strong, such diffusion behavior will yield a change in signal attenuation that will be reflected in tensor diffusivities. **(C)** If, however, some diffusing molecules cease to interact with microstructural barriers during a given diffusion time, the axial to radial relationship would be dependent on the local geometry leading to heterogeneous tensor behaviors across the brain. **(D)** In the extreme case, where the diffusion time is too short for molecular displacements to be hindered, tensor diffusivity measures would be unable to detect further change. Large *b*-values—enabling long diffusion times and strong gradients—make the diffusion measurement with magnetic resonance both more sensitive to subtle microstructural alterations in highly restricted environments, and less prone to diffusion-time dependencies.

A further caveat is that most DTI experimental designs assume that tensor behaviors are independent of diffusion time—i.e., that the brain parenchyma is a restricted environment in a “pseudo-Gaussian” state, where diffusion time is long relative to the time needed for water molecules to be hindered/restricted by cellular membranes or other microstructural components. Note that the former are thought to be the primary microstructural restrictions to water diffusion in white matter (Beaulieu and Allen, 1994). This can be generally assumed to be valid because in typical clinical situations, diffusion times range between 30 and 50 ms, which translate to *in vivo* 1D molecular displacements of 13–17 μm. Such displacements are an order of magnitude greater than, for example, the typical axon diameter in the corpus callosum of young individuals, which is approximately 1 μm (Aboitiz et al., 1992)—though axons can also be much larger. In lay terms, the reason this is important is that in order to know that restrictions of water diffusion due to barriers in neural tissue are present, one must measure long enough “diffusion paths” to ensure water molecules have had a chance to “bounce off” some barriers. Figure 5A illustrates this scenario, where such paths are long relative to the distance between physical obstacles, enabling DTI to sense differential bulk behaviors parallel and perpendicular to white matter tracts. In patients with neurodegenerative diseases, however, cellular membranes may become more permeable; or the extra-cellular space might be greater due to axonal loss, demyelination, and/or glial pathology. Such effects—alone or in combination—may result in local environments where, during a given diffusion time, water molecules interact only partially with microstructural boundaries. Figure 5C illustrates such a scenario where some paths (e.g., the blue path) do not encounter any obstacle during a given diffusion time, i.e., they behave as if they were in an unrestricted medium. In theory, during disease progression the loss of large myelinated axons, or clumps of adjacent tracts, may result in diffusion paths that cannot capture the full extent of such “voidage.” This is the so-called “quasi-restricted” regime, in which measurements depend on diffusion time—i.e., if water molecules were allowed to diffuse twice as far by doubling the diffusion time, microstructural boundaries would be sensed again, resulting in smaller radial diffusivity relative to axial diffusivity, hence leading to a more anisotropic tensor. Such behavior may inadvertently compromise whole brain assessments because the same neurodegenerative process, e.g., axonal loss, could impact diffusivities differentially across WM tract environments with different axon size distributions, packing arrangements or degrees of myelination. Suppose, for instance, an enlargement of the extra-cellular space of 20% due to axonal loss; its impact on RD would be different, for example, in a scenario where axons are lost sparsely than if a small bundle of large myelinated axons degenerate together. In the latter case, if molecular displacements were small relative to the “new” boundaries, FA would be underestimated as a result. It may be desirable to detect such diffusion-time dependencies (Baron and Beaulieu, 2014); but they imply that whole brain analyses would be biased toward such phenomena if they were proven to be present in some diseased regions but not in others. Furthermore, there might be scenarios of extreme degeneration, or CSF contamination in a voxel, where the majority of water molecules behave as in an unrestricted medium; under such circumstances, further loss of microstructural integrity would lie largely undetected with DTI because water mobility would not reflect the presence of any boundary (as in Figure 5D).

While these important theoretical considerations and limitations should not be ignored, DTI can be very useful to probe abnormal tissue microstructure. For instance, in a pseudo-Gaussian scenario where several fiber tracts are damaged as a consequence of a disease, diffusion hindrance would be reduced (see Figure 5B), i.e., *pdf*s would approximate more readily to a Gaussian distribution, and would return greater diffusivity values closer to the intrinsic unrestricted self-diffusion coefficient; it is this change in diffusivity pattern that can indicate abnormality in degenerative disease states. In addition, because the tensor model yields three rotationally invariant—and orthogonal—diffusivities (λ_1_, λ_2_, and λ_3_), a disproportionate diffusivity change along a particular orientation will impact on absolute (axial and radial) diffusivities, or composite measures of diffusion anisotropy such as FA as illustrated in Figure 3.

It is presently unknown whether diffusion-time dependencies might be a confounding force in the study of neurodegenerative diseases; for now, they remind us that caution must be at the forefront in DTI interpretations. Unfortunately, DTI studies in neurodegenerative diseases have often been guilty of over-interpreting results, particularly when such interpretations are based on consistency with prior knowledge rather than offering a proof of a given mechanism. Mechanisms underpinning DTI changes in clinical cohorts are often inferred by citing homology with controlled animal experiments or computer simulations, where processes such as myelin loss, fiber reorganization, changes in membrane permeability, *etc*, are modeled in isolation—for a extended review see Beaulieu (2002). Reductionist approaches such as animal experiments are important in that they can highlight the types of neural changes that are possible to detect with DTI but there is a potentially serious error in logic when such observations are exported directly to explain changes observed in human neurodegeneration. For instance, if a given DTI change has been observed in an animal model of demyelination and then the same DTI change is observed in a degenerative disease, one will often read that the mechanism in the latter is also demyelination. The error in logic is that there are only a finite number of ways in which the diffusion tensor can change, and for any given change, it is incorrect to assume that only one mechanism might be the explanation—there may be many different types of pathological change that could cause the same diffusion tensor alteration. Using a clinical analogy, this is the same error in logic as stating that because sub-arachnoid hemorrhage can cause headache, any person with a headache must have a sub-arachnoid hemorrhage. Furthermore, it seems highly unlikely that a single mechanism will dominate the diffusion weighted MR signal because fiber degradation processes such as Wallerian degeneration—which typically involve the complex combination of cytoskeleton dissolution, contraction, fragmentation, disintegration of myelin and glial disposal (Coleman and Freeman, 2010)—are unlikely to occur simultaneously across all cells in a single measurement voxel. Such complexity, worsened by the diffusion regime uncertainty, suggests that DTI results should not be interpreted beyond their canonical form—i.e., that increased (or reduced) diffusivities are caused by less (or more) restrictive barriers to water diffusion, and that diffusion anisotropy alterations are driven by orientation dependent changes in the configuration of such microstructural arrangement. What these exactly represent in neurobiological terms can only be speculated. Nevertheless, DTI-derived diffusivity and anisotropy measurements can offer important mechanistic insights toward understanding the sequence of events that lead to neurodegeneration by highlighting tracts that are diverging from normality, even if diffusion MRI, at present, cannot precisely state what the underlying pathology driving such divergence might be.

## Technical considerations for clinical DTI studies

Historically, T_1_-weighting has been the MRI contrast of choice to study structural abnormalities in the human brain. It is clear that imaging parameter discrepancies or different field strengths result in differential method sensitivity, but overall they tend to be relatively concordant if the same processing steps are used. This is because structural MRI post-processing methods typically concentrate on resolving and standardizing different types of tissue using large amounts of prior anatomical knowledge; this makes such analysis strategies relatively immune to differences, e.g., in signal-to-noise ratio (SNR). Unlike structural MRI, however, DTI relies on *quantitative* information to yield meaningful assessments; thus, DTI sensitivity and stability strongly depend on how robustly the signals have been acquired. Therefore, additional precaution must be taken when reviewing the literature because the ability of diffusion MRI to accurately reconstruct tensorial information strongly depends on measurement SNR, which varies throughout the brain, between subjects and across studies. It is likely that when non-specialists read an imaging study, acquisition details—which may seem an impenetrable paragraph of technical jargon—are skipped over. Such information though is critical to DTI because some acquisition protocols that have been applied in clinical studies are simply not fit for purpose. It is beyond the scope of the present manuscript to discuss in detail every relevant factor—for a more specialized review, see e.g., Jones and Cercignani (2010)—but to help readers to critically evaluate the literature in this ever-growing field, a number of the most clinically relevant technical considerations will be briefly discussed:

### The DTI acquisition scheme

The “*b*-value” is the parameter that tunes how much microstructural information the diffusion MR signal can carry. Relatively large *b*-values are needed to ensure both (i) that strong diffusion gradients sensitize water motion in complex, highly restricted environments, and (ii) that the diffusion time is sufficiently long to enable “meandering” diffusion paths to probe less restricted (or diseased) microstructure. Early computer simulations suggested that for a single non-zero *b*-value experiment, *b* = 1250 s/mm^2^ minimizes fitting errors for a mean diffusivity of 0.7 × 10^−3^ mm^2^/s, i.e., approximately that of brain parenchyma (Xing et al., 1997). More recent simulations have demonstrated that even taking the simplification that the human brain is constituted by single-orientation fiber populations, i.e., that crossing fibers are not present, the *b*-value—if interested in MD and FA—should not be lower than *b* = 900 s/mm^2^ (Alexander and Barker, 2005). Such results highlight a pitfall with a large number of studies—particularly at 1.5 Tesla (T)—that used a maximum *b*-value of 700 s/mm^2^. Capturing high order signal decays reflecting non-Gaussian restricted diffusion with a smaller-than-optimal single non-zero *b*-value translates to inaccurate “fits,” which, as illustrated in Figure 4B, may lead to tensor reconstructions that do not accurately reflect the true diffusion process, and hence, the underlying microstructure. The use of larger *b*-values—closer to optimal for tensor estimation in WM—can help reduce such residual errors, but high *b*-values further attenuate the MR signal; thus, in order to ensure that diffusion-weighted signals are well above the “noise floor,” a common practice is to take advantage of the higher baseline SNR at stronger magnetic fields (e.g., 3T MRI). It should be noted, however, that stronger fields are not always favorable for DTI because the diffusion-weighted signal is also T_2_ dependent, and T_2_ is shorter for stronger fields, which, in turn, translates to faster signal decays. This is undesirable, and poses a serious challenge, particularly at ultra-high field (7T and above), because whole brain DTI acquisitions typically consist of a single-shot echo planar imaging (EPI) pulse sequence with long-lasting diffusion and readout gradients resulting in long delays before the signal can be acquired, i.e., long echo time. At 3T, however, the gain in baseline SNR usually overcomes the T_2_ penalty; that is why presently 3T MRI is the platform of choice for diffusion tensor neuroimaging. It is noteworthy, looking to the future, that low SNRs will become less of a problem for clinical studies with the advent of high performance gradient systems capable of stronger field gradients with shorter switching times, which enable the use of higher *b*-values with shorter echo times.

Returning to the present, it is important to avoid low SNR because the noise floor may damp signal decay, resulting in artefactually reduced diffusivities; this effect is orientation dependent in the WM because faster signal decays—caused by greater diffusivities along tracts—are more vulnerable to this effect, resulting in artefactual reductions of diffusion anisotropy. This is critically important because if one is attempting to map the distribution of WM change in degenerative brain disease it is possible to miss changes in affected areas where the local fiber orientations were more vulnerable to the effects of low SNR. Signal levels are modulated by the magnetic field strength, the type of radio-frequency coil used and imaging parameters such as the echo time (TE); number of *b*-values (N_b_); number of repeat excitations (NEX); receiver bandwidth or parallel imaging acceleration, e.g., GRAPPA (Griswold et al., 2002) or SENSE (Pruessmann et al., 1999). Unfortunately, however, SNRs are not usually reported in the literature. In addition, details about the number of channels available in phased-array coils, acceleration factor or receiver bandwidth are also often not reported in clinical studies, making comparisons between works hard to interpret from methods sections.

The influence of suboptimal gradient sampling schemes is also an important factor to consider when scrutinizing the literature. Diffusion weighting along six unique orientations (see Figures 2A,B) and the acquisition of a reference non-diffusion weighted (or b_0_) image are the minimum requirements to reconstruct DTI parametric maps; but to ensure primary diffusivities are independent of fiber orientation—i.e., to ensure DTI metrics are rotationally invariant –, one has to probe diffusion behaviors evenly and at a high spatial frequency. This is because tensor eigenvalues, hence diffusivities and anisotropy estimates, are more robust when WM tracts are collinear with one of the motion-sensitizing gradients (Jones, 2004). It is, therefore, essential that large numbers of diffusion-encoding orientations (N_d_) are used for accurate DTI measurements in whole brain studies. In fact, the minimum number for robust tensor reconstruction has been estimated to range between *N*_d_ = 20 (Papadakis et al., 2000) and *N*_d_ = 30 directions (Batchelor et al., 2003; Jones, 2004), though a recent computer simulation has shown that a number of directions as low as *N*_d_ = 12 can yield accurate MD estimates if the number of *b*-values is sufficiently large, e.g., *N*_b_ = 5 (Correia et al., 2009). Interestingly, such robust behavior with respect to noise could not be replicated using repeat measures (NEX = 5) with a single *b*-value. This work, therefore, highlights the importance of collecting more than one *b*-value—untrue for NEX > 1—both to sensitize the DTI measurement to a wider range of diffusivities—i.e., microstructural environments—and to reduce the measurement bias due to noise. In clinical research practice, however, the number of measurements, N_m_, is typically limited to 60–70 for a total scan time of 10 min; hence an optimal compromise between N_d_ and N_b_ must be achieved. Correia et al.'s DTI simulation revealed that for *N*_m_ = 60 measurements, if the interest is in MD, *N*_d_ = 12 × N_b_ = 5 is the optimal set-up—i.e., a large number of *b*-values is preferred. For FA estimation, however, a larger number of directions—N_d_ ≥ 30 (as in Batchelor et al., 2003; Jones, 2004)—is needed to establish accurate axial-to-radial diffusivity relationships, whereas little gain was observed for *N*_b_ > 2. Therefore, an important outcome of these studies is that *N*_d_ = 30 × N_b_ = 2 appears to be a good compromise to ensure that tensor reconstructions are reliable; leading to the notion that the majority of clinical studies—typically sensitized with a single *b*-value and often, *N*_d_ ≤ 15 with NEX ≥ 2—may have led to poor measurement stability.

An additional factor that may play a role in DTI robustness is the accurate estimation of *S*_0_ through multiple b_0_ scans (N_*b*0_). To our knowledge, this has not been systematically investigated, though in theory its impact will depend on SNR (TE, parallel acceleration factor, *etc*), and on the prescribed number of *b*-values—becoming less relevant with increasing N_b_. It is conceivable, however, that a large N_*b*0_ can help stabilize the measurements for little additional scan time—particularly in single *b*-value experiments.

**Figure 6:**
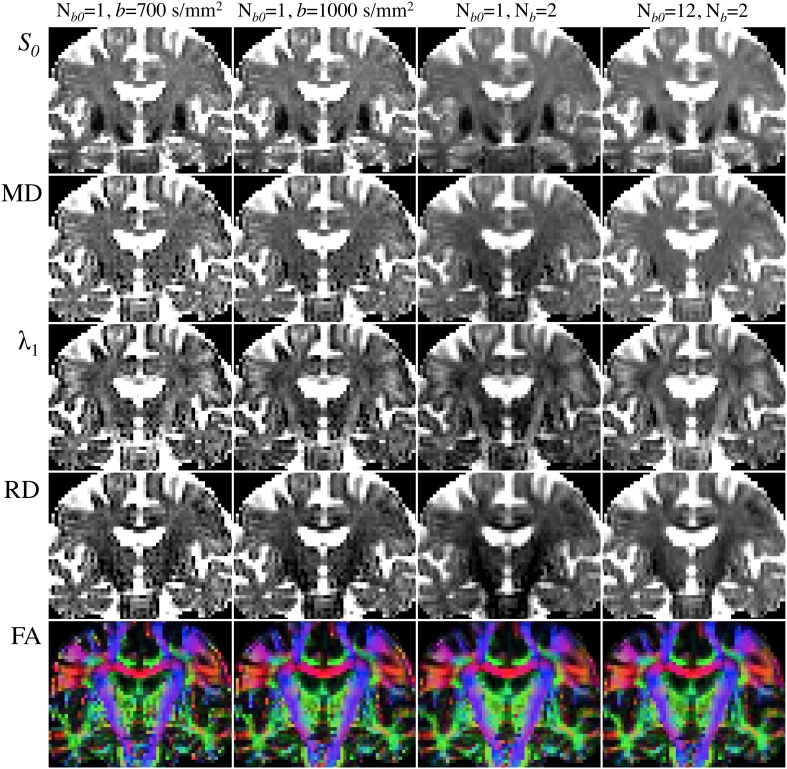
**Comparison of DTI parametric maps with different *b*-value, number of *b*-values (N_b_) and number of b_0_ scans (N_*b*0_)**. MRI measurements were performed on a Siemens Verio 3T system (Siemens Medical Systems, Erlangen, Germany)—gradient coils capable of 45 mT/m and 200 T/m/s slew rate—with a 32-channel phased-array head-coil. Diffusion volumes were acquired using a standard twice-refocused, single-shot EPI pulse sequence: repetition/echo time = 9000/94 ms; matrix, 120 × 120; 63 contiguous slices aligned parallel to the anterior commissure/posterior commissure line; voxel size: 2 × 2 × 2 mm^3^; 7/8-phase partial Fourier; bandwidth of 1667 Hz/pixel and echo spacing of 0.68 ms. Diffusion gradients were applied along *N*_d_ = 30 non-collinear directions (Siemens default vectors) with *N*_b_ = 2 non-zero *b*-values (*b* = 700 and 1000 s/mm^2^), and *N*_*b*0_ = 12 reference scans. Parallel imaging was enabled (GRAPPA, acceleration factor = 2 and 38 reference lines), leading to a total scan time of 11 min and 15 s. DTI maps were computed with standard tools from FSL's diffusion toolbox. (Left to right columns): (i) *N*_b_ = 1 (*b* = 700 s/mm^2^), *N*_*b*0_ = 1 (5:06); (ii) *N*_b_ = 1 (*b* = 1000 s/mm^2^), *N*_*b*0_ = 1 (5:06); (iii) *N*_*b*_ = 2 (*b* = 700 and 1000 s/mm^2^), *N*_*b*0_ = 1 (9:36); (iv) *N*_b_ = 2 (*b* = 700 and 1000 s/mm^2^), *N*_*b*0_ = 12 (11:15).

Figure 6 illustrates the choice of *b*-value, N_b_ and N_*b*0_ dependence on DTI reconstructions. All maps were inferred from subsets (or the complete dataset) of a single acquisition with *N*_d_ = 30, two non-zero *b*-values (*b* = 700 and 1000 s/mm^2^) and *N*_*b*0_ = 12—i.e., *N*_m_ = 72. Notable improvement in the calculation of diffusivities and anisotropy can be observed with the increase in *b*-value from 700 to 1000 s/mm^2^. As would be expected, further improvements are noticeable when using the information from both *b*-values in combination; though the highest diffusivity-to-noise ratios are returned by the complete dataset with a larger number of b_0_ scans. It is, therefore, clear that they all contribute to improving DTI measurement stability.

**Figure 7:**
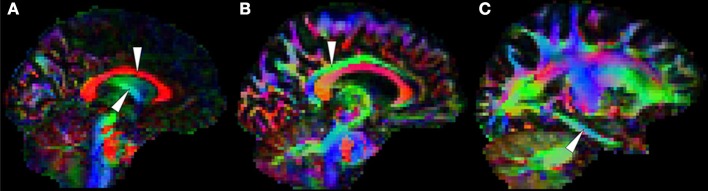
**White matter tracts that are usually prone to measurement error**. **(A)** The body of the corpus callosum (top arrow) and the body of the fornix (bottom arrow) are often problematic because they are adjacent to cerebrospinal fluid. The cingulum bundle, **(B)** which is typically less vulnerable at the level of the posterior cingulate, **(C)** is sometimes prone to partial volume contamination and other types of measurement error in the parahippocampal region due to its thinner physical appearance (Jones et al., 2013a) and its closer proximity to rostral temporal areas known to be affected by magnetic susceptibility artifacts. Note in addition that orbitofrontal white matter is also vulnerable to such artifacts.

Correia et al. (2009) showed that measurements from suboptimal schemes quickly deviate from true simulated values with decreasing SNR; but SNR is not only dictated by the factors enumerated above, but also by image resolution. One finds a plethora of voxel sizes in the published DTI literature. In the best case scenario, images are composed of isometric 2 × 2 × 2 mm^3^ voxels, but a large number of studies—particularly early research at 1.5T—used slices more than twice as thick as in-plane resolution to ensure sufficient measurement SNR. Non-isometric voxels, however, are undesirable for volumetric analyses because they encourage volume averaging along the slice direction, which translates to spurious direction-dependent error biases (Oouchi et al., 2007). An alternative would be to encode smaller matrices with larger isotropic voxels, which is common practice, but one should not ignore that partial volume effects caused by large measurement voxels can also be highly detrimental in DTI due to GM and/or CSF contamination. This, in turn, can lead to systematic errors in patient populations because typically patients have more brain atrophy than controls, and therefore, DTI alterations may reflect CSF inclusions rather than true microstructural changes. It should be noted that the WM tract of interest in the present Research Topic—the fornix—due to its close vicinity to ventricular CSF is particularly vulnerable to this phenomenon as illustrated in Figure 7.

In conclusion, although DTI is becoming a mature technique, there is not yet a universal agreement on the minimum requirements for a reliable DTI acquisition, or on an optimal image acquisition scheme for a given scan time, so we must reconcile with the fact that different DTI studies to date may have sensitized their acquisitions very differently.

### The post-processing methodology

**Figure 8:**
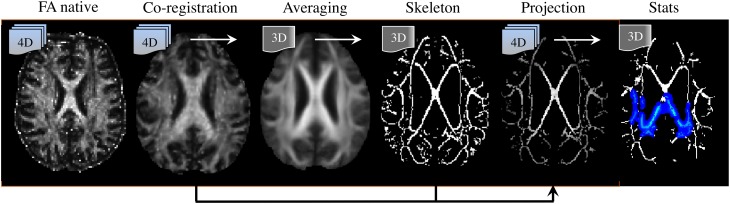
**Tract-based spatial statistics (TBSS) processing pipeline**. (Left to right) DTI-derived fractional anisotropy (FA) images are co-registered to a template; they are then averaged, from which a “skeleton” containing all major tract centers common to all subjects is derived. Skeleton voxels with low *FA*-values (typically *FA* < 0.2) are excluded to ensure only white matter is present. Next, spatially normalized FA images are projected to the skeleton. In this step, the center of each tract is identified for each individual FA image, and projection vectors to their analogous location in the skeleton are computed and applied. Transformation fields (to template space) and projection vectors (to the skeleton) are then also applied to the additional DTI parametric maps (i.e., MD, λ_1_, RD). Finally, non-parametric, permutation based, statistical testing of the null-hypothesis is performed.

Post-processing methods are also highly relevant to interpreting DTI literature. To date, a number of analysis strategies have been used including region-average, histogram or voxel-/cluster-based. No method, however, has demonstrated systematic superiority in every scenario; though it is widely accepted that for unbiased whole-brain assessments, the tract-based spatial statistics (TBSS) approach (Smith et al., 2006) has advantages and is the most desirable technique for volumetric DTI analysis at present (see Figure 8). TBSS—part of FMRIB's software library or FSL (Smith et al., 2004)—enables whole brain assessment of WM tract integrity in neurodegenerative diseases using DTI data without the need for *a priori* hypotheses about the spatial location of degenerative involvement. TBSS circumvents the lack of anatomical landmarks in WM, which is a limiting factor for manual tracing of tracts of interest in native space. TBSS deals with this problem by automatically co-registering all DTI parametric maps to a standard template; also, unlike histogram analyses, it can resolve the specific location of abnormal clusters; it is not biased by the dependency issues known to affect tractography based regional analysis (Besseling et al., 2012); and finally, TBSS does not require convolution with a smoothing kernel, as is used in standard VBA methods, due to the introduction of a skeletonisation step. The skeleton is derived from averaging FA images across all subjects and subsequent identification of tract centers; this ensures the analysis is carried out exclusively on definite WM. But the main advantage of projecting DTI data to a WM skeleton is that it corrects, to some extent, co-registration inaccuracies, rendering the data more comparable than in VBA. In addition, performing statistics only along the center of major WM bundles minimizes the effects of partial volume contamination and reduces the number of statistical tests. Furthermore, TBSS' non-parametric statistical method does not require normally distributed data and, as for any randomization approach, it is inherently robust against Type-I errors (Winkler et al., 2014).

The default TBSS processing pipeline, however, suffers from its own sources of inaccuracy and limitations: it fails to rotate the **B**-matrix after realigning all images to account for motion and eddy-current effects (Leemans and Jones, 2009); it implements a fast—but oversimplified—tensor fitting routine (Jones and Cercignani, 2010) that assumes single fiber populations in each voxel, hence leading to potential miscalculations in areas of crossing and kissing fibers (Jbabdi et al., 2010); its non-linear co-registration algorithm, FNIRT, performs moderately relative to other *state-of-the-art* methods (Klein et al., 2009); assessments are spatially restricted to WM tract centers only—ignoring, therefore, a large amount of WM^1^; and finally, it suffers from spatial- and orientation-dependent statistical sensitivity (Edden and Jones, 2011). That is, TBSS is not completely unbiased. Such limitations should not be ignored; in fact, they suggest that in certain scenarios other methods might be preferable. For example, the fornix is often excluded from the WM skeleton due to the perennial issue of image misregistration (Keihaninejad et al., 2012) or does not survive correction for multiple comparisons at the cluster level due to its small physical size (Acosta-Cabronero et al., 2012; Schwarz et al., 2014). The fornix, however, has clear boundaries in FA maps (at least through the midline), and more advanced co-registration methods exist; thus, although FNIRT-based TBSS studies in AD will be the focus here to homogenize the potential confounding error introduced by different post-processing routines and because, to date, it is the most widely used method of whole-brain DTI analysis, it should be highlighted that manual tracing or automated regional extraction using high-performing, tensor-orientation enabled (Zhang et al., 2006) or intensity-based, diffeomorphic image registration algorithms (Avants et al., 2008) might be better suited to study structures such as the fornix.

Turning to the issue of image misregistration with standard FSL tools, a recent study has compared the warping performance for a number of co-registration strategies, including the standard TBSS procedure of warping each subject to the FMRIB's FA template (ST-TBSS), the previously recommended method of using the most representative subject as an intermediate registration step (RS-TBSS), and building study-specific (SS-TBSS) or group-wise templates (GW-TBSS)—all in the context of AD (Keihaninejad et al., 2012). The lack of ground truth makes the visual assessment of TBSS results in AD hard to interpret; however, using inter-subject variability and a proxy-measure of false positive rate as indicators of overall warping performance, Keihaninejad et al.'s results suggest that RS-TBSS is substantially inferior to the other methods, and that the use of the GW-TBSS approach might be beneficial.

### The subject cohort

An important factor that must be considered is the nature of the patient cohort under investigation. Many studies focus on the prodromal stages of AD—typically patients diagnosed with subjective or mild cognitive impairment (SCI/MCI), genetically at risk (e.g., carriers of the apolipoprotein E ε 4 allele), and/or those who are positive for an AD biomarker, e.g., CSF analysis or amyloid-ligand PET. It is noteworthy, however, that not all risk indicators are equivalent; while amyloid-PET is an unambiguous marker of AD neuropathology (Clark et al., 2012), CSF markers are not as robust due to inconsistent standardization (Verwey et al., 2009). In addition, genetic risk profiles (for sporadic AD) are problematic because they are only risk factors. Most troublesome, though, are studies that report cross-sectional cohorts of SCI or MCI with neither biomarker nor longitudinal outcome data to confirm symptoms were due to early AD. Cohorts without such information are impossible to interpret because of the potentially large number of false positive cases meeting such inclusion criteria (Jicha et al., 2006). This problem can be further compounded if imaging studies stratify MCI into amnestic (aMCI), non-amnestic (naMCI) and multi-domain (mdMCI), or, into “early” and “late” MCI without outcome or biomarker data. Individuals belonging to an mdMCI or late MCI group, being more cognitively impaired, have a higher probability of having AD, while naMCI has a lower probability (Mitchell et al., 2009). These are, however, only probabilities and do not indicate the pathology of each individual in a group. If the aim is to study the neurobiology—as it should be in an imaging study—of prodromal AD, a subject with naMCI due to AD is an appropriate inclusion whereas one with mdMCI that is not due to AD pathology is not. The present review, therefore, will not discuss such studies unless outcome data through clinical follow-up, or amyloid-PET/CSF analysis, confirmed that subjects in these pre-dementia stages were likely to have had incipient AD. It should be noted that meeting established criteria for probable AD (McKhann et al., 1984) does not completely predict AD neuropathology at autopsy, but the risk of including false positive cases is far lower than for SCI/MCI.

A further confounding effect that must always be considered is the comorbidity of WM hyperintensities. Studies typically deal with this by excluding cases with such lesions through visual inspection of T_2_-weighted images; this can also be supported by semi-quantitative measures using the Fazekas visual rating scale (Fazekas et al., 1987). It should be highlighted, however, that there is no consensus on what constitutes an unacceptable level of lesion burden for a DTI analysis in dementia although such information is very relevant given the extremely high prevalence of WM hyperintensities in the target population. This would be an interesting topic for further research, as would more automated and quantifiable measures of lesion load. For instance, it seems reasonable that subjects with negligible amounts of WM signal change would still be appropriate for inclusion in a DTI study, but this immediately raises the question of how one defines “negligible.” More objective scales and a consensus on an acceptable threshold would, therefore, be desirable.

### Multi-center study designs

Multi-site designs are becoming increasingly common in AD research, particularly with the advent of the Alzheimer's Disease Neuroimaging Initiative (ADNI) and related spin-offs (Jack et al., 2010). Whilst such initiatives incorporate a pilot stage to estimate measurement stability across centers, a systematic assessment of inter-site DTI variability has not yet been carried out within the ADNI framework. An independent European initiative, however, performed such measurements and found significant FA variations across scanners, leading to the conclusion that FA variability cannot be fully controlled by the use of identical scanner type and acquisition parameters (Teipel et al., 2011). This result highlights that, at least until full cross-validations confirm otherwise, multi-center studies should account for potentially confounding scanner effects—a practice that is not commonly carried out. It should be noted, therefore, that multi-center studies in the present review have been included only to confirm widely reproduced observations, but little emphasis has been given to idiosyncratic results generated from such datasets.

### Literature synthesis: inclusion criteria

In an effort to evaluate the DTI literature to date in AD, studies meeting the “essential” criteria for this review (Table 1) were selected. Table 2 illustrates the iterative process that led to identification of 13 publications in which an acceptable acquisition and analysis protocol was employed; and in which patients had either clinically probable AD, MCI-stage AD where longitudinal follow-up or biomarkers were used to define probable AD status, or patients with autosomally inherited known gene mutations for AD (Acosta-Cabronero et al., 2010, 2012; Douaud et al., 2011; Bosch et al., 2012; Huang et al., 2012; Canu et al., 2013; Fieremans et al., 2013; Mahoney et al., 2013; Nir et al., 2013; Rowley et al., 2013; Ryan et al., 2013; Lim et al., 2014; Molinuevo et al., 2014). It should be stressed that Table 2 highlights the order of iterations by which studies were excluded; it should not be read to mean that for each criterion the number of excluded studies equals that number of studies in total that failed on that specific criterion because many studies failed on multiple criteria. Table 1 also includes a list of proposals of additional criteria for future studies based on current knowledge.

**Table 1:** **Selection criteria for DTI studies in Alzheimer's disease included in the present review and additional guidelines for future studies**.

**Essential (inclusion criteria for this review)**
**DTI ACQUISITION**
✓ Number of diffusion-encoding directions equal to or greater than 30
✓ Quasi-isometric voxels—ratio between in-plane resolution and slice thickness greater than 0.75, i.e., the geometry of 1.875 × 1.875 × 2.5 mm^3^ voxels is at the lower limit
✓ Voxels smaller than 20 mm^3^, i.e., image resolution 2.7 × 2.7 × 2.7 mm^3^ is at the upper limit
✓ Maximum *b*-value equal to or greater than 900 s/mm^2^
**STUDY COHORT**
✓ (i) Clinical diagnosis of probable AD (at acquisition or through clinical follow-up), (ii) positive CSF analysis for AD (iii) positive amyloid-PET, or (iv) autosomal dominantly inherited mutation carriers
✓ Number of subjects equal or greater than *N* = 10 in each group
**DATA ANALYSIS**
✓ Voxel-/cluster-wise (or region-average) whole-brain TBSS
✓ Show results for both FA and MD (at least)
✓ Demonstrate results are robust against multiple testing effects
**Desirable (additional suggested criteria for future studies)**
**DTI ACQUISITION**
+ Studies performed on a single scanner
+ Use of multiple *b*-values, i.e., *N_b_*≥ 2
+ If N*_b_* = 1, total number of measurements greater than 60, i.e., N_m_ > 60
+ Ensure stable *S*_0_ measurement, i.e., N_*b*0_ ≥ 5
+ Voxels equal to or smaller than 8 mm^3^, i.e., image resolution 2 × 2 × 2 mm^3^ or finer
+ Perfect voxel symmetry (or within 90%)
**DATA ANALYSIS**
+ Avoidance of RS-TBSS pipelines

**Table 2:** **Summary of the hierarchical iterative steps leading to the selection of 13 DTI studies in Alzheimer's disease**.

	**# of studies**	**Remaining**
1. PubMed search (“diffusion tensor” OR DTI) AND (Alzheimer’s) on 5/8/2014	476	
2. Not relevant (i.e., tractography based structural/functional connectivity studies, DTI works with marginal reference to AD, *etc*)	285	191
3. Not TBSS (i.e., VBA, ROI, atlas-based, *etc*)	118	73
4. TBSS in MCI or ApoE4 carriers without clinical outcome or additional biomarker	27	46
5. TBSS in AD using images with unacceptably thick scan slices	11	35
6. TBSS in AD using an insufficient number of diffusion-encoding directions (i.e., *N*_d_ < 30)	10	25
7. TBSS in AD using low maximum *b*-value (i.e., *b*_max_ < 900 s/mm^2^)	4	21
8. TBSS in non-representative groups (i.e., *N* < 10 AD subjects)	2	19
9. TBSS to study AD-related aspects not directly relevant to this comparison (e.g., investigation of neural correlates)	2	17
10. TBSS in AD with a non-conventional statistical approach	1	16
11. TBSS in AD where no contrast against controls was shown	1	15
12. TBSS in AD where only the FA contrast was shown	2	13
Selected TBSS studies in AD	13	

Details of the 13 studies that were identified from the literature review can be found in the Supplementary Table. It should also be noted that several of the studies (Douaud et al., 2011; Bosch et al., 2012; Huang et al., 2012; Nir et al., 2013; Rowley et al., 2013) included both AD and MCI groups where the latter did not meet entry criteria for the present review. Only data from the AD groups is included from such studies.

## TBSS in AD: findings from the literature overview

After careful literature filtering for potential technical problems in past studies, a number of consistent TBSS behaviors were identified; it would be wrong to assume, however, that such results provide a definitive understanding of WM changes in AD because, as discussed, alterations in structures that are prone to error may lie undetected for technical reasons. In contrast, prominent DTI effects that have been reproduced in a number of methodologically sound studies are evidence of real phenomena and that may, thus, be interpreted with scientific rigor as disease-related processes.

### What does the distribution of diffusion tensor alterations tell about AD?

**Figure 9:**
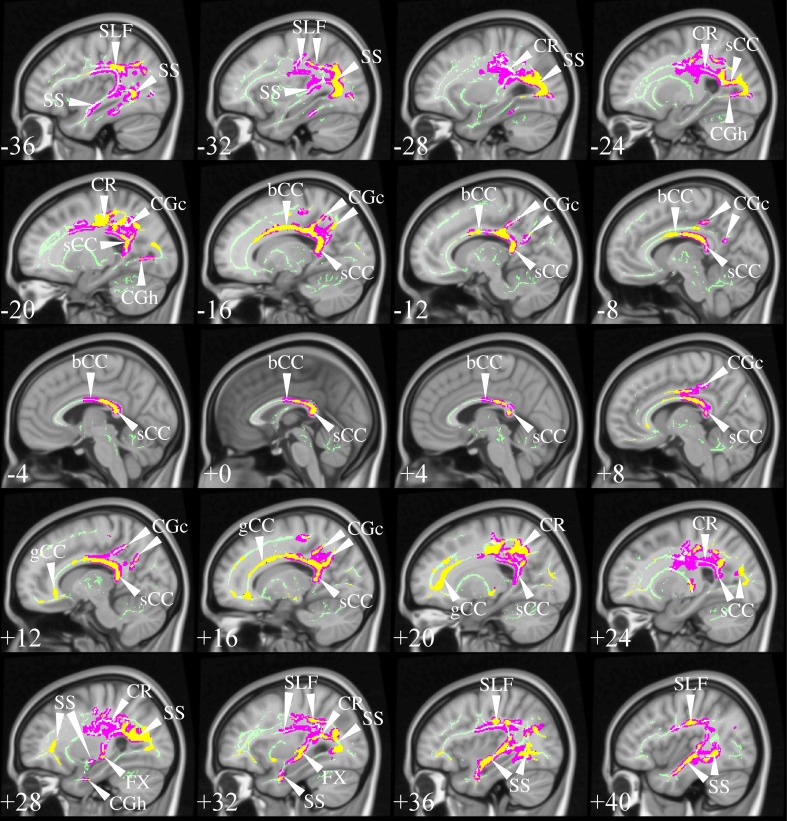
**TBSS in AD**. TBSS results for *N* = 43 early-stage AD patients (age: 70 ± 6, <MMSE > = 24 ± 4) vs. *N* = 26 matched controls (age: 68 ± 6) (Acosta-Cabronero et al., 2012). Pink clusters denote increased MD in patients, whereas those in yellow represent FA reductions at *P* < 0.05 enabling threshold free cluster enhancement (TFCE) (Smith and Nichols, 2009) and controlling the family-wise error (FWE) rate. Thresholded statistical maps are overlaid onto the TBSS skeleton and MNI152 template. Sagittal MNI coordinates are given in millimeter where *x* < 0 is left. Abbreviations: [SLF] superior longitudinal fasciculus; [SS] sagittal stratum; [CR] corona radiata; [s/b/gCC] splenium/body/genu of the corpus callosum; [CGc/h] cingulum at the level of the posterior cingulate/parahippocampus; and [FX] fornix.

The selection process identified TBSS studies in AD that generally had a consistent common denominator—i.e., widespread and confluent tensor abnormalities in parietal, temporal and pre-frontal WM—specifically involving long association fibers (including limbic tracts) and inter-hemispheric connections through the corpus callosum. Generally speaking changes were more apparent in posterior areas compared to frontal areas as would be expected from prior knowledge of cortical atrophy studies and FDG-PET. Figure 9 exemplifies such a distribution in a mild AD cohort (Acosta-Cabronero et al., 2012), where the posterior corpus callosum, cingulum bundle at the level of the posterior cingulate gyrus, superior longitudinal fasciculus and sagittal stratum (including the inferior longitudinal fasciculus, fronto-occipital fasciculus, and posterior thalamic radiation) were affected. Additional involvement is also commonly found in superior temporo-parietal WM at the level of the corona radiata and centrum semiovale—composed of intertwined long/short association and projection tracts.

Two exceptions, however, were found; the studies by Ryan et al. (2013) and Lim et al. (2014) did not find any cross-sectional differences in pre-symptomatic, autosomal dominant (PSEN1) mutation carriers and a group of MCI-stage AD patients defined by CSF biomarkers, respectively. The group reported by Ryan et al. (2013) were estimated to be a mean of 5.6 years from symptom onset so it simply may be that the group was too early to have major changes in WM; to this end it was notable that as a group there was also no significant hippocampal atrophy. The group only comprised *N* = 10 mutation carriers which also suggests it may have been underpowered to detect subtle changes with whole brain TBSS analysis; finally, the control group used was significantly older than the PSEN1 group which may have also masked abnormalities. The cohort in Lim et al. (2014) were *N* = 16 early MCI-stage AD subjects scanned under the umbrella of the ADNI2 framework, i.e., on 14 different scanners, which may, thus, have contributed to the lack of sensitivity. Though one might be tempted, in light of these results, to conclude that DTI is unable to detect WM changes in such early stages, the *post-hoc* regional study carried out by Ryan et al. (2013); the TBSS study by Molinuevo et al. (2014) in a group of cognitively normal and SCI subjects (positive for CSF biomarkers); and an atlas-based regional study (with suitable methods) in cognitively normal (amyloid-PET positive) subjects (Racine et al., 2014) suggest otherwise, as these reported DTI alterations that were consistent with the distribution in Figure 9.

The results from Canu et al. (2013) are also worthy of further discussion. The study examined two AD cohorts with different onset ages, but matched otherwise for disease severity, with two appropriately age-matched control groups. The early-onset group showed very widespread abnormalities highly concordant with Figure 9, but that spread slightly more anteriorly along association tracts and the genu of the corpus callosum. These were moderately impaired cohorts, i.e., average mini-mental cognitive examination (MMSE) score (Folstein et al., 1975) around 20/30; thus it would be reasonable to expect that changes had extended more extensively into frontal areas. Interestingly, however, the result in the late-onset cohort was relatively insensitive. Whilst differences were found in posterior areas largely overlapping the clusters in Figure 9, the distribution was markedly less extensive, which was interpreted as less severe damage than in the earlier-onset group of patients. While this might be true, the study highlights another potentially important confound; that is, the age of the control group. The mean age of controls for the contrast with “late-onset” AD was 73 years but at this age one would expect around one third of a control population to be amyloid positive, i.e., in the pre-clinical stage of AD (Villemagne et al., 2011). In contrast, the control group for the early-onset AD cohort had a mean age of 59 years—an age at which one would expect negligible, if any, contamination from pre-clinical AD. In other words, older control subjects are probabilistically more likely to introduce undesirable variability into group comparisons, which may explain some of the differences in sensitivity across studies. This suggests that when targeting “late-onset” AD, it would be desirable in future that the control group was defined by being amyloid-negative, in addition to being cognitively intact, to ensure that this possible confound was not attenuating sensitivity.

Studies in symptomatic, young PSEN1 carriers (Ryan et al., 2013), or in relatively young sporadic AD cohorts (age: 63 ± 5, Mahoney et al.), where control groups were young and abnormalities, very extensive—with posterior predilection but spreading toward anterior areas—also support the notion that control age might be a contributing factor affecting analysis sensitivity. However, extensive abnormalities are not a unique feature of young cohorts, a number of studies in older groups have also shown a similar behavior, e.g., mild AD in Fieremans et al. (2013), mild AD in Acosta-Cabronero et al. (2012), mild AD in Douaud et al. (2011), mild AD (ADNI data) in Rowley et al. (2013) and mild AD (ADNI data) in Nir et al. (2013). In such studies, the posterior parietal and superior temporal regions highlighted in Figure 9 were key features, but abnormalities also spread more anteriorly along association tracts and also included extensively the anterior corpus callosum, corticocortical association fibers running through the external capsule and the anterior limb of the internal capsule. Disentangling whether more, or less, widespread distributions across studies are the result of subtle technical factors, differences in disease severity, in control variability, or in cohort size, is impossible to disambiguate, but it should be highlighted that from the selected studies, cohorts with the most widespread abnormality patterns had, in general, more advanced disease stages than the cohort displayed in Figure 9, suggesting disease severity is a dominant factor in result sensitivity. A recent study in two age-matched AD cohorts with different disease severity that controlled for all the above factors using the same acquisition, the same control cohort and the same number of subjects in each patient group, showed evidence indicating a posterior first, then more anterior distribution in more advanced disease stages (Acosta-Cabronero et al., 2012)—matching the expected progression of degeneration as would be measured by cerebral glucose metabolism (Minoshima et al., 1997; Nestor et al., 2003b). In summary, although a number of additional factors might have contributed to variability in result sensitivity across studies, disease staging appears to drive the overall distribution of WM abnormalities in AD.

Focusing on the earliest DTI changes in AD specifically, Molinuevo et al. (2014) studied a combined cohort of cognitively normal subjects and SCI with a CSF profile consistent with AD, while Acosta-Cabronero et al. (2012) reported on patients with MCI-stage AD confirmed through longitudinal follow-up. The two studies showed a consistent picture of the affected neural network in incipient stages of AD. Both results highlighted involvement of parietal and superior temporal WM bilaterally—spatial distributions that are also in close agreement with the expected landscape of WM involvement that can be inferred from the pattern of glucose hypometabolism in very mild AD (Nestor et al., 2003b, 2006).

### Differential diffusion metric sensitivity during the course of AD

An interesting by-product of TBSS analyses in AD has been in showing that alterations in diffusion behavior are complex—while early (and some recent) studies tended to focus only on FA—often leading to largely insensitive results (Damoiseaux et al., 2009; Zarei et al., 2009; Balachandar et al., 2014)—, more extensive abnormality distributions for MD have been a consistent finding across studies (Acosta-Cabronero et al., 2010, 2012; Douaud et al., 2011; Huang et al., 2012; Fieremans et al., 2013; Mahoney et al., 2013; Nir et al., 2013; Rowley et al., 2013; Ryan et al., 2013). What this behavior represents in neurobiological terms, however, is unknown because, as discussed already, DTI offers limited information about the geometry of the underlying damaged microstructure. In contrast, what DTI enables us to infer from such behavior is that WM degeneration in AD results in alterations to WM tissue that are not grossly driven along any preferential direction (Acosta-Cabronero et al., 2010). Subtle orientation dependent behaviors missed by FA can be explored by dissecting the apparent diffusion coefficient encapsulated in MD into its primary components, i.e., λ_1_ (also commonly referred to in the literature as AxD or DA) and RD. A superficial reading of studies examining these primary components appear not to provide a clear picture—a number of studies such as those in very mild AD by Acosta-Cabronero et al. (2012) and in CSF-positive SCI by Molinuevo et al. (2014) indicate that λ_1_ might be the dominant source of DTI abnormalities; whereas other studies such as those in mild AD by Mahoney et al. (2013) and Bosch et al. (2012), and in moderately impaired AD by Ryan et al. (2013) show greater RD effects. Douaud et al. (2011) proposed that the λ_1_ increase could be explained as an artifact of loss of one fiber population in areas of crossing fibers (see Figure 3). This would be a plausible theory if increased λ_1_ were restricted to areas of crossing fibers (e.g., centrum semiovale), however increased λ_1_ in AD is not limited to such regions: the same phenomenon is observed in the midline corpus callosum and fornix where crossing tracts are not present. This indicates that increased λ_1_ in AD must be due to another mechanism but what that might be in biological terms is, at present, unclear^2^.

The patient cohorts in Mahoney et al. (2013); Bosch et al. (2012) and the symptomatic cohort in Ryan et al. (2013), which all reported increased RD, had more advanced dementia (MMSE < 23) than those outlined with extensive λ_1_ changes, suggesting that on closer inspection of the literature, individual component metrics of the diffusion tensor may evolve along different trajectories with disease progression. This implies that different metrics may be more or less sensitive to different disease stages. This hypothesis was tested on two—very mildly (MCI-stage) and mildly impaired—AD groups using the same MR acquisition and post-processing methods (Acosta-Cabronero et al., 2012). The distribution of increased λ_1_ involving posterior temporo-parietal WM—largely consistent with Molinuevo et al. (2014)—was identified as the first sign of change. In the more advanced group, however, increased RD—which, in turn, drove FA reductions—emerged in these regions that had shown increased λ_1_ in the very mild group. As degeneration spread to new areas—notably frontal and left temporal WM—increased λ_1_ was again the first abnormality to appear. These results suggested that while λ_1_ increase is the first sign of change in a given location, RD is relatively insensitive at the earliest disease stages but becomes progressively more abnormal as the disease progresses. Confirming this theory, a subset of AD patients in the same study, who were scanned serially, revealed that RD/FA changes emerged at follow up in areas where increased λ_1_ had been seen at baseline. Interestingly, λ_1_ increases did not progressively worsen over time with longitudinal follow-up. There was, furthermore, no evidence for progressive worsening of λ_1_ between the two severity stages in the cross-sectional cohorts when investigating the midline splenium in isolation. In fact, if anything there was a slight tendency for the increase in λ_1_ to lessen with advancing disease (Acosta-Cabronero et al., 2012); in other words suggesting that when a WM region first becomes affected in AD, there is a sharp increase in λ_1_, that may possibly attenuate with further disease progression.

In summary, RD progressively increases (and therefore FA progressively decreases) as a function of disease severity—as defined by degree of cognitive impairment—whereas λ_1_ does not progressively increase. That increasing λ_1_ is not a function of disease severity, at least in the midline splenium, suggests that the early λ_1_ increase in AD may be capturing an upstream event to axonal degeneration, whereas processes more directly related to neuronal loss might dominate RD/FA dynamics—implying thus that, whilst λ_1_ in the splenium could act as a state-specific marker in prodromal disease stages, the spatial distribution of RD/FA changes may be a more suitable staging biomarker.

### Fornix changes in AD

**Figure 10:**
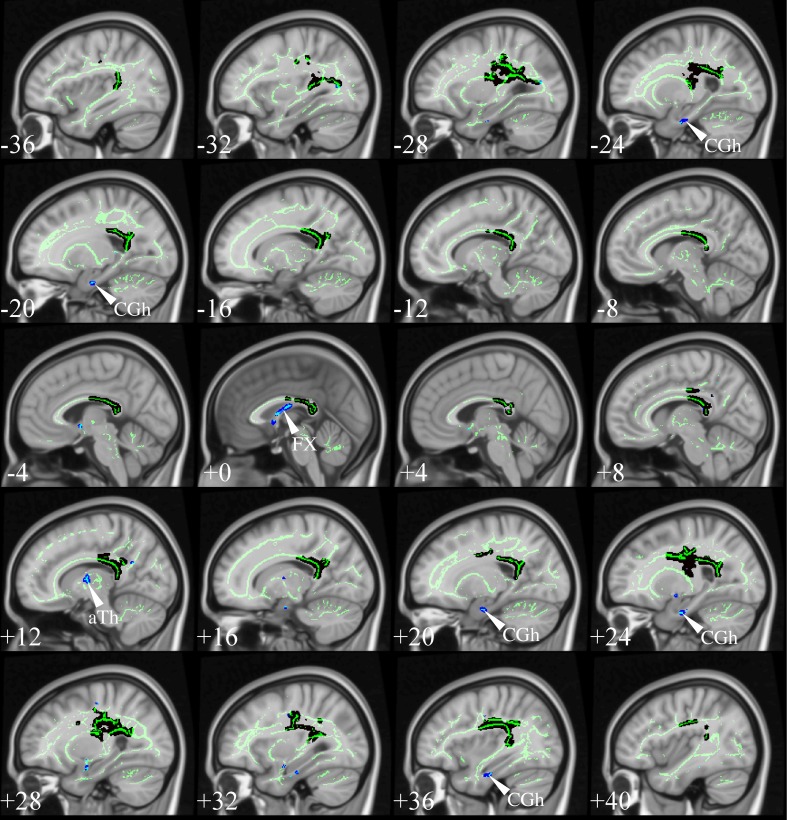
**TBSS in very mild AD**. TBSS results for *N* = 21 very mild AD patients (age: 72 ± 5, <MMSE > = 26 ± 2) vs. *N* = 26 matched controls (age: 68 ± 6) (Acosta-Cabronero et al., 2012). The patient group included *N* = 16 subjects who were scanned with a diagnosis of mild cognitive impairment but were subsequently shown to have probable AD with longitudinal follow-up. Black clusters denote increased λ_1_ in patients at P_FWE_ < 0.05, whereas those in blue represent changes with *post-hoc* control over the false discovery rate (FDR) at *q* < 0.05. Thresholded statistical maps are overlaid onto the TBSS skeleton and MNI152 template. Sagittal coordinates are given in millimeter (*x* < 0 is left). Abbreviations: [aTh] anterior thalamic white matter; [CGh] cingulum at the level of the hippocampus; and [FX] fornix.

Although atlas-based approaches consistently find strong effects in the fornix (Keihaninejad et al., 2013; Racine et al., 2014), this was not, however, a universal feature of the TBSS studies reviewed above. Such tracts are often excessively penalized due to their small size by the strictness of non-parametric statistics controlling for family-wise error (FWE) rate. Using, however, the more lenient false-discovery rate (FDR) correction (Benjamini and Hochberg, 1995) to show abnormalities that are less prominent at the cluster level—yet statistically robust—fornix changes emerge also with TBSS analysis. Figure 10 illustrates the impact of this slight statistical modification, resulting in “new” λ_1_ abnormalities in the fornix, parahippocampal WM and anterior thalamus (possibly capturing change in the mammillo-thalamic tract). These structures are not just random new blobs—they precisely target major WM connections in the limbic-diencephalic network that has been highlighted as the early hypometabolic landscape of AD (Nestor et al., 2003b, 2006). For a focused review on the specific role of the fornix in this network, including the possibility of it being therapeutic target for deep brain stimulation in AD, see the review in this Research Topic (Oishi and Lyketsos, 2014).

The notion that limbic tracts and the corpus callosum—particularly the splenium—are key features in early stages of AD has now a solid body of evidence. The theory that these regions should show the most advanced degeneration in the course of AD (and therefore be the first to show RD/FA changes) when other areas, being less advanced in the course of disease, would only show λ_1_ changes is strongly supported by the regional TBSS analysis in mild AD carried out by Huang et al. (2012). These authors found λ_1_ increase in association and projection tracts and RD/FA effects in the corpus callosum, cingulum and fornix, precisely suggesting that the latter were at a more advanced stage of degeneration compared to the former. In a similarly impaired cohort extracted from the ADNI dataset, Nir et al.'s regional TBSS study also found strong RD/FA alterations for such structures. In addition, a prospective study—targeting specifically the corpus callosum, fornix and cingulum—found significant cross-sectional and longitudinal tensor differences for all three structures (Keihaninejad et al., 2013). The common aspect in these studies is that—consistent with all the selected works—absolute diffusivities were increased in AD relative to controls, whereas FA was reduced.

## Conclusion

In summary, the existing literature supports the theory that a bilateral neural network that involves preferentially the cingulum bundle, fornix and corpus callosum is vulnerable to early disease processes triggered by the Alzheimer's disease neuropathological cascade. This suggests that the nodes that this network inter-connects—i.e., the mesial temporal lobe, mammillary bodies, thalamus, and posterior cingulate—degenerate as a network, rather than in isolation.

To conclude with a note of caution and a window to future research directions. While much effort was made to rationalize published DTI studies in AD for this review, it remains possible that a large number of technical factors including suboptimal acquisitions, poor tensor reconstruction routines, poor image co-registration performance, or intrinsic DTI limitations, to name a few, might still have resulted in some systematically incorrect results across the literature, in turn, leading to incompletely valid interpretations in this review. A number of technical developments, however, should hopefully soon help confirm, or reject, the above theories: stronger gradient systems enabling larger *b*-values, shorter echo times and smaller voxels; improved image quality through multiband acquisitions (Frost et al., 2014); the prescription of multiple *b*-values; the implementation into standard tensor reconstruction pipelines of distortion correction, **B**-matrix rotation and multi-component fitting routines with a free-water component (Pasternak et al., 2009); the application of advanced diffusion models beyond the single tensor such as diffusion kurtosis imaging (Jensen et al., 2005), NODDI (Zhang et al., 2012), CHARMED (Assaf and Basser, 2005), or q-space approaches such as q-ball reconstruction (Tuch, 2004) or diffusion spectrum imaging (Wedeen et al., 2005); the use of *state-of-the-art* co-registration methods (Zhang et al., 2006; Avants et al., 2008); and a long *et cetera*.

### Conflict of interest statement

The authors declare that the research was conducted in the absence of any commercial or financial relationships that could be construed as a potential conflict of interest.

## Footnotes

^1^In practical terms, however, this is not a problem for neurodegenerative diseases in which degeneration involves large-scale networks as opposed to tiny focal lesions. Visual inspection of a skeleton typically demonstrates that TBSS does not systematically miss any large chunks of the brain. In fact it can be desirable, particularly in neurodegenerative diseases, where the data reduction step to tract centers eliminates to a greater extent the vulnerability to partial volume contamination due to focal lesions.

^2^One possible situation where loss of crossing fibers may explain increased λ_1_ was a study that examined the neural substrate of topographical memory impairment in AD (Pengas et al., 2012). The study in question found correlations between increasing λ_1_ and decreasing task performance; interestingly the regions that correlated were all in close proximity to the cortical ribbon—in other words, in the U-fiber layer. It is tempting, therefore, to speculate that local degeneration of GM, in turn meaning less axons entering adjacent WM, might lead to a simplification of the adjacent U-fiber layer—i.e., less crossing fibers—making increased λ_1_ correlate significantly.
