# Supplementary material for: Diffusion tensor imaging in Alzheimer's disease: insights into the limbic-diencephalic network and methodological considerations
Source: Front Aging Neurosci. 2014 Oct 2;6:266. doi: 10.3389/fnagi.2014.00266 (PMC4183111; doi:10.3389/fnagi.2014.00266)
Supplement: Supplementary file 1 [file Table1.PDF]

Supplementary Table. Summary of methods for the DTI studies in Alzheimer's disease that met the 'essential' inclusion criteria in Acosta-Cabronero and Nestor, *"Diffusion tensor imaging in Alzheimer's disease: insights into the limbic-diencephalic network and methodological considerations"*, Frontiers in Aging Neuroscience. Highlighted blocks signify specific methodological aspects that fail to meet our recommendations (as 'desirable') for future studies.

	DTI Acquisition													Study Cohorts							Data Analysis
														Control		AD					
	**Field**	**# of sites**	N_{b0}	N_b	**b-value** (s/mm^2)	N_d	**NEX**	N_m	**TE** (ms)	**TR** (s)	**Resolution** (mm^3)	V_{voxel} (mm^3)	**Voxel ratio**	**N**	**age**	**N**	**age**	**MMSE**	**Status**	**Basis**	**Method**
Acosta-Cabronero *et al.* (2010)	3T	1	**1**	**1**	1000	63	1	64	90	7.8	2×2×2	8	1	13	67(6)	25	70(6)	23(4)	AD	Clinical follow-up	**RS-TBSS**
Douaud *et al.* (2011)	3T	1	6	**1**	900	30	2	66	89	7.0	**2.5×2.5×2.5**	**16**	1	61	71(8)	53	74(9)	24(3)	AD	Clinical diagnosis	SS-TBSS
Acosta-Cabronero *et al.* (2012)	3T	1	**1**	**1**	1000	63	1	64	90	7.8	2×2×2	8	1	26	68(6)	21	72(5)	26(2)	MCI/AD	Clinical follow-up	ST-TBSS
														26	68(6)	21	69(6)	22(4)	AD	Clinical follow-up	ST-TBSS
Bosch *et al.* (2012)	3T	1	**1**	**1**	1000	30	1	**31**	89	5.7	2×2×2	8	1	15	75(6)	15	72(6)	21(3)	AD	Clinical diagnosis	ST-TBSS
Huang *et al.* (2012)	3T	1	**2**	**1**	1000	30	2	62	97	7.8	2×2×2.2	9	0.91	24	70(7)	26	71(8)	23(2)	AD	Clinical diagnosis	ST-TBSS (ROI)
Canu *et al.* (2013)	3T	1	**1**	**1**	900	35	1	**36**	58	8.8	**1.87×1.87×2.3**	8	**0.81**	16	73(4)	35	75(5)	20(4)	AD	Clinical diagnosis	ST-TBSS
														24	59(3)	22	59(4)	19(5)	AD	Clinical diagnosis	ST-TBSS
Fieremans *et al.* (2013)	3T	1	11	2	1000/ 2000	30	2	131	96	5.9	**2.7×2.7×2.7**	**20**	1	15	78(4)	14	78(10)	22(6)	AD	Clinical diagnosis	ST-TBSS
Mahoney *et al.* (2013)	3T	1	9	**1**	1000	64	2	137	91	6.8	**2.5×2.5×2.5**	**16**	1	20	65(6)	20	63(5)	21(5)	AD	Clinical diagnosis	SS-TBSS
Nir *et al.* (2013)	3T	**14**	5	**1**	1000	41	1	**46**	63	9.0	**2.7×2.7×2.7**	**20**	1	44	73(6)	23	76(10)	23(2)	AD	Clinical diagnosis	ST-TBSS (ROI)
Rowley *et al.* (2013)	3T	**14**	5	**1**	1000	41	1	**46**	63	9.0	**2.7×2.7×2.7**	**20**	1	25	73(6)	15	76(11)	23(2)	AD	Clinical diagnosis	ST-TBSS
Ryan *et al.* (2013)	3T	1	9	**1**	1000	64	2	137	91	6.8	**2.5×2.5×2.5**	**16**	1	20	44(13)	10	38(5)	29[28-30]	Healthy	PSEN1 mutation	GW-TBSS
														20	44(13)	10	49(9)	19[13-24]	AD	PSEN1 mutation	GW-TBSS
Lim *et al.* (2014)	3T	**14**	5	**1**	1000	41	1	**46**	63	9.0	**2.7×2.7×2.7**	**20**	1	20	75(5)	16	74(8)	27(2)	MCI	Amyloid PET/CSF	ST-TBSS
Molinuevo *et al.* (2014)	3T	1	**2**	**1**	1000	30	2	62	89	7.6	2×2×2	8	1	19	69(6)	19	70(8)	28(2)	Healthy/ SCI	CSF	ST-TBSS
